# Evaluation of dual-wavelength excitation autofluorescence imaging of colorectal tumours with a high-sensitivity CMOS imager: a cross-sectional study

**DOI:** 10.1186/s12876-015-0339-6

**Published:** 2015-09-02

**Authors:** Yoko Kominami, Shigeto Yoshida, Shinji Tanaka, Rie Miyaki, Yoji Sanomura, Min-Woong Seo, Keiichiro Kagawa, Shoji Kawahito, Hidenobu Arimoto, Kenji Yamada, Kazuaki Chayama

**Affiliations:** 1 Department of Gastroenterology and Metabolism, Graduate School of Biomedical and Health Science, Hiroshima University, Hiroshima, Japan; 2 Department of Endoscopy and Medicine, Graduate School of Biomedical and Health Science, Hiroshima University, Hiroshima, Japan; 3 Research Institute of Electronics, Shizuoka University, Shizuoka, Japan; 4 Electronics and Photonics Research Institute, National Institute of Advanced Industrial Science and Technology (AIST), Tsukuba, Japan; 5 Graduate School of Medicine, Division of Health Sciences, Osaka University, Osaka, Japan

**Keywords:** Dual-wavelength excitation autofluorescence image, Colorectal tumour, High-sensitivity CMOS imager

## Abstract

**Background:**

It is important to devise efficient and easy methods of detecting colorectal tumours to reduce mortality from colorectal cancer. Dual-wavelength excitation autofluorescence intensity can be used to visualize colorectal tumours. Therefore, we evaluated dual-wavelength excitation autofluorescence images of colorectal tumours obtained with a newly developed, high-sensitivity complementary metal-oxide-semiconductor (CMOS) imager.

**Methods:**

A total 107 colorectal tumours (44 adenomas, 43 adenocarcinomas with intramucosal invasion, and 20 sessile serrated adenoma/polyps [SSA/Ps]) in 98 patients who underwent endoscopic tumour resection were included. The specimens were irradiated with excitation light at 365 nm and 405 nm, and autofluorescence images measured with a 475 ± 25-nm band pass filter were obtained using a new, high-sensitivity CMOS imager. Ratio images (F365ex/F405ex) were created to evaluate the lesion brightness compared with that of normal mucosa, and specimens were categorized into a no signal or high signal group.

**Results:**

Adenomas and adenocarcinomas were depicted in 87 ratio images, with 86.2 % (*n* = 75) in the High signal group. SSA/P was depicted in 20 ratio images, with 70.0 % (*n* = 14) in the High signal group.

**Conclusions:**

Dual-wavelength excitation autofluorescence images of colorectal tumours can be acquired using our high-sensitivity CMOS imager, and are useful in detecting colorectal tumours.

## Background

Colorectal tumours are among the most common tumours worldwide [1]. Because most adenomas are premalignant lesions, elimination of colon adenoma is an effective strategy for preventing colon cancer development, and several studies have reported that endoscopic resection of colon adenoma decreases colon cancer mortality. Additionally, serrated colorectal lesions are considered precursors for up to one-third of colorectal cancers. Sensitive detection of adenomas would contribute to risk prediction and to the planning of appropriate surveillance intervals, especially because the number of adenomas is a good determinative factor for predicting the long-term risk of advanced neoplasia [2–4]. However, systematic reviews of serial colonoscopy using auto-fluorescence imaging (AFI) and high-resolution white light colonoscopy showed that 15–32 % of colorectal adenomas, particularly flat and depressed adenomas such as sessile serrated adenomas, were missed by colonoscopy [5, 6].

Fluorescence emission, also known as autofluorescence, is an intrinsic property of cells that is caused by endogenous fluorophores. Research on autofluorescence and its clinical applications has been conducted in various fields [7–11]. Current knowledge suggests that the composition and bio-distribution of fluorophores such as collagen, porphyrin, nicotinamide adenine dinucleotide hydrogenase (NADH), flavin adenine dinucleotide, and lysosome granules in the colorectal wall may be responsible for the visible differences within the tissue [9–18].

Autofluorescence imaging (AFI) captures the fluorescence emitted from intestinal and other tissues. The AFI device produces and delivers excitation light at 442 nm to the tissue surface and then captures the reflected light and emitted fluorescence using high-sensitivity charge-coupled devices (CCDs) [6, 19, 20]. AFI primarily captures the fluorescence emitted by collagen in the submucosal layer, and abnormal areas are indicated by decreased fluorescence intensity [19]. This suggests that AFI images the tumour indirectly, and furthermore, AFI is disadvantaged by its high false-positive rate and low specificity of 35 % [21]. Thus, technical developments allowing tumour autofluorescence to be directly observed are expected.

In a recent study, the dual-wavelength excitation method, using excitation wavelengths of 365 nm and 405 nm and a 470 ± 20-nm band pass filter, was employed to measure autofluorescence; the study found that this technique was useful for visualizing and discriminating colonic adenomas on cross-section [22]. In addition to the direct capture of tumour autofluorescence images, the brightness difference between the lesions and normal mucosa was also suggested to reflect the amount of NADH, which is a known metabolism-related fluorophore.

For imaging, the weak autofluorescence necessitates a long exposure time when using a normal-sensitivity CCD image sensor, or it requires a high-sensitivity EM-CCD image sensor that must be cooled to at least −20 °C to reduce noise. A new high-sensitivity image sensor that does not require cooling is needed for practical clinical use. We recently developed a high-sensitivity complementary metal-oxide-semiconductor (CMOS) imager that can be used at room temperature [23, 24].

In the present study, we evaluated the surface autofluorescence in human colorectal adenoma, and adenocarcinoma, and sessile serrated adenoma/polyp (SSA/P) specimens using a dual-wavelength excitation method with the newly developed high-sensitivity CMOS imager. We also evaluated the relationship between the brightness of colonic tumours during autofluorescence imaging and their histologic structure.

## Methods

### Specimen preparation

We examined 107 specimens obtained from 98 patients who underwent endoscopic mucosal resection or endoscopic submucosal dissection of one or more colorectal lesions. The patients were treated at Hiroshima University Hospital between October 2012 and March 2014, and included 62 men and 36 women aged 65.1 ± 11.3 years (mean ± standard deviation [SD]).

To minimize metabolic biotransformation after resection, the specimens were immersed in phosphate-buffered saline solution immediately after removal, and autofluorescence images were obtained as soon as possible. The time between endoscopic resection and image capture was less than 5 min in all cases.

The lesions were classified morphologically according to the Paris endoscopic classification of superficial neoplastic lesions [25]. Forty-four of 107 lesions were diagnosed as adenoma, 43 as adenocarcinoma with intramucosal invasion, and 20 as SSA/P.

This study was conducted with the approval of the ethics committee of Hiroshima University Hospital. Informed consent was obtained from all patients and/or family members for endoscopic examination and pathologic examination of tissue samples.

### Acquisition of autofluorescence images

The surface autofluorescence of colorectal tumours was evaluated using the dual-wavelength excitation method [22]. A stereomicroscope (SZX12; Olympus Medical Systems Corp., Tokyo, Japan) for microscopic autofluorescence analysis was equipped with an objective lens (DF PLAPO 1.2× PF2; Olympus) and a band pass filter (475 nm/25×; Edmund Optics, Barrington, NJ, USA) [23, 24]. An LED light (UVLED illuminator, spot type, 365 nm and 405 nm, Kenko Tokina, Tokyo, Japan) served as the excitation light source. The newly developed CMOS imager was designed to be highly sensitive with low noise and a wide dynamic range. The CMOS imager was fabricated in 0.18-μm technology at the following specifications: 1.3 megapixels (1284 [H] × 1028 [V]); pixel size, 7.1 μm × 7.1 μm; and maximum frame rate, 30 frames/s [23, 24]. Autofluorescence images were obtained with the CMOS imager at room temperature.

**Fig. 1:**
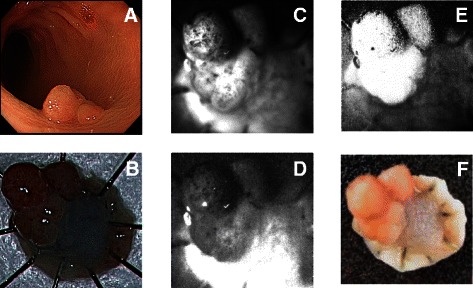
Representative autofluorescence ratio image. Autofluorescence images were obtained with a high-sensitivity CMOS imager by irradiating the resected specimen with 405-nm followed by 365-nm excitation light. The ratio images were then created by dividing the F365ex (365 nm) by F405ex (405 nm). **a**. Colon tumour observed during endoscopy. **b**. Fresh resected specimen. **c**. Autofluorescence image at 365-nm excitation (F365ex). **d**. Autofluorescence imaging at 405-nm excitation (F405ex). **e**. The calculated ratio image. **f**. Resected formalin-fixed specimen

Sequential autofluorescence surface images of specimens containing both tumour and normal tissue were obtained using a band pass filter (475 ± 25 nm) at a 365-nm excitation (F365ex) followed by a 405-nm excitation (F405ex), as shown in Fig. 1.

#### Creation and evaluation of dual-wavelength excitation images

**Fig. 2:**
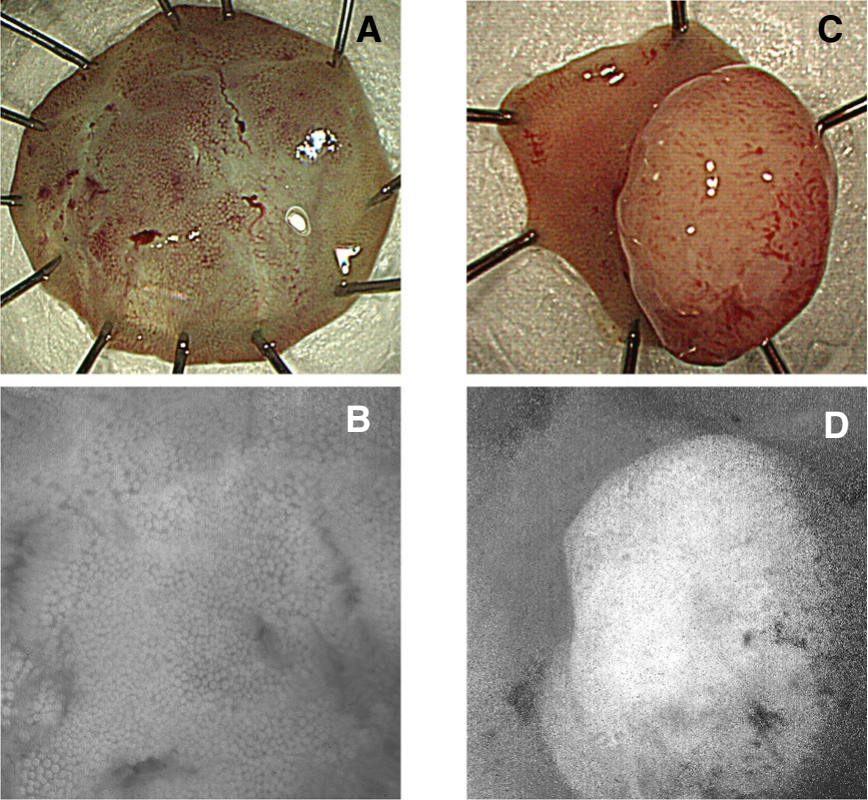
Classification of lesion brightness in autofluorescence ratio images. **a**. Fresh resected specimen. **b**. No-signal group: In comparison to normal mucosa, the lesion does not appear bright (ratio image of **a**). **c**. Fresh resected specimen. **d**. High-signal group: In comparison to normal mucosa, the lesion appears bright (ratio image of **c**)

The autofluorescence signal intensities were calculated from the acquired images. Autofluorescence ratio (F365ex/F405ex) images were constructed from the paired autofluorescence images acquired with 365-nm excitation and 405-nm excitation (Fig. 1). The lesion and normal tissue brightness on the ratio image were compared and categorized as either no signal intensity (no-signal group) or high signal intensity (high-signal group) (Fig. 2) by a gastroenterologist who was blinded to the histologic findings.

#### Interobserver agreement

Another gastroenterologist categorized the same ratio images as high-signal group and no-signal group. Interobserver agreement between the two gastroenterologists was then analysed to determine kappa values.

#### Statistical analysis

The interobserver agreement of the classification of the ratio images, i.e., the agreement between two gastroenterologists, was tested using the kappa statistic. Differences in the autofluorescence ratio between the tumour types were analysed by the Fisher’s exact test. All statistical analyses were performed using the R environment for statistical analyses (https://www.r-project.org/), with *P* < 0.05 considered statistically significant.

## Results

The tumour autofluorescence on the ratio images according to the histologic type is detailed in Table 1. Adenomas and adenocarcinomas were depicted in 87 ratio images, with 86.2 % (*n* = 75) in the High signal group. SSA/P was depicted in 20 ratio images, and 70.0 % (*n* = 14) were in the High signal group.

**Table 1:** Autofluorescence ratio images of colorectal tumours according to histologic type

	No signal	High signal	Total
Adenoma and adenocarcinoma	12 (13.8)	75 (86.2)	87 (100)
SSA/P	6 (30.0)	14 (70.0)	20 (100)
Total	18 (16.8)	89 (83.2)	107 (100)

Data presented as the number (percentage) of specimens

SSA/P, sessile serrated adenoma/polyp

*p* = 0.0997

We further evaluated the appearance of the adenomas and adenocarcinomas and SSA/Ps according to the macroscopic type. Macroscopic non-polypoid-type adenomas and adenocarcinomas were depicted in 32 ratio images, with 78.1 % (*n* = 25) in the High signal group, while polypoid-type tumours were depicted in 55 images, with 90.1 % (*n* = 50) in the High signal group (Table 2).

**Table 2:** Autofluorescence ratio images of adenoma and adenocarcinoma according to macroscopic type

	No signal	High signal	Total
Non-polypoid type	7 (21.9)	25 (78.1)	32 (100)
Polypoid type	5 (9.9)	50 (90.1)	55 (100)
Total	12 (13.8)	75 (86.2)	87 (100)

Data presented as the number (percentage) of specimens

*p* = 0.115

Macroscopic non-polypoid type SSA/P was depicted in 12 ratio images, with 58.3 % (*n* = 7) in the High signal group, and polypoid-type tumours were depicted on eight images, with 87.5 % (*n* = 7) in the High signal group (Table 3).

**Table 3:** Autofluorescence ratio images of SSA/P tumours according to macroscopic type

	No signal	High signal	Total
Non-polypoid type	5 (41.7)	7 (58.3)	12 (100)
Polypoid type	1 (12.5)	7 (87.5)	8 (100)
Total	6 (30.0)	14 (70.0)	20 (100)

Data presented as the number (percentage) of specimens

SSA/P, sessile serrated adenoma/polyp

*p* = 0.325

Regarding the interobserver variability of the ratio images, the kappa value between two gastroenterologists was 0.902, and the agreement was excellent.

## Discussion

Generally, colonoscopy is performed to diagnose and treat colorectal tumours. Effective prevention of colon cancer requires that diagnostic modalities both detect and differentiate between benign lesions and those requiring treatment [26–29]. With the development of endoscopy, clinicians are able to image colorectal lesions in detail, including microscopic changes; however, the level of sensitivity relies on the skill and experience of the operator. Therefore, it is important to develop methods to easily detect colorectal tumours.

Matsuda et al. proposed the superiority of AFI for detecting polyps in the proximal colon compared with white light endoscopy using a modified back-to-back method [30]. However, another study reported that, though highly sensitive at 99 %, AFI also had a low specificity of 35 % for the diagnosis of neoplastic and non-neoplastic colorectal polyps [21]. Hence, despite the increased adenoma detection rate and high sensitivity in differentiating between neoplastic and non-neoplastic colorectal polyps, the clinical utility of AFI has been limited by its low specificity, which results in a high false-positive rate for colorectal neoplasia diagnosis. Narrow-band imaging was reportedly comparable to chromoendoscopy for distinguishing between neoplastic and nonneoplastic polyps [31–37], but it is unable to accurately differentiate between sessile serrated adenomas and non-neoplastic lesions, as 75 % of sessile serrated adenomas were undiagnosed according to one report [21].

Using the dual-wavelength excitation method [18], fluorescence ratio (F365ex/F405ex) images were taken of colorectal polyps with an EM-CCD imager and band pass filter. The images clearly distinguished adenomatous lesions with distinct borders and adenomas. In our study, 86.2 % of adenomas and adenocarcinomas were clearly depicted on autofluorescence images obtained with our newly developed, high-sensitivity CMOS imager. On histopathologic analysis, 70.0 % of SSA/Ps and 86.2 % of adenomas and adenocarcinomas on the ratio images were categorized in the High signal group. Our findings suggest that it is possible to detect not only adenomas and adenocarcinomas but also SSA/Ps by the dual-wavelength excitation method. By contrast, Imaizumi et al. reported that the differences in signal intensities were insufficient to distinguish normal mucosa and hyperplastic polyps [21]. This suggests that the discrepancy in fluorescence reflects histologic differences between hyperplastic polyps, SSA/Ps, and adenomas and adenocarcinomas. SSA/Ps and adenomas are premalignant lesions, and SSA/Ps are the principal serrated precursors for colorectal cancer, while hyperplastic polyps are not [38]. Although further study is needed to enable diagnosis of the metabolic status of colorectal tumours based on the autofluorescence intensity, the present results indicate that autofluorescence imaging may be able to detect SSA/Ps and adenomas and adenocarcinomas, and differentiate these neoplasms from hyperplastic polyps.

We observed colorectal superficial areas that fluoresced upon light excitation. Most specimens of the adenoma and adenocarcinoma and SSA/P were categorized in the High signal group, and no significant difference was observed according to macroscopic type, *i.e.* non-polypoid and polypoid types. Our study suggests that autofluorescence may be useful for visualizing colorectal tumours regardless of the macroscopic type, but additional study of many more cases is needed. The interobserver agreement of the classification of the ratio images could be an issue; however, in our classification, excellent agreement was observed, with high kappa values. However, there was a limitation to this study in that it was performed ex vivo; thus, we did not evaluate the clinical utility of endoscopic imaging for detection and differentiation of colonic polyps. Further in vivo studies should be performed when our dual-wavelength excitation autofluorescence endoscopy technique, which operators can easily perform in real-time, is developed. Clinical application of dual-wavelength excitation autofluorescence endoscopy requires incorporating a high-sensitivity image sensor into the endoscope. Most gastrointestinal endoscopes are equipped with a normal range sensitivity CCD image sensor. Therefore, we developed a new CMOS image sensor with high sensitivity, low noise, and a wide dynamic range at room temperature [22, 23, 39]. It may be possible to incorporate this technology into endoscopes.

In our study, both adenomas and adenocarcinomas and SSA/Ps can emit light using the dual-wavelength excitation method, which suggests that inexperienced operators can easily identify lesions using this technique without oversight.

## Conclusions

Although continued study is needed, we conclude that surface autofluorescence images of colorectal lesions can be obtained with our newly developed high-sensitivity CMOS imager. Furthermore, our study indicates that dual-wavelength excitation autofluorescence imaging is indeed useful for the clinical detection of colorectal tumours.

## References

[1] Hamilton SR, Aaltonen LA (2000). World Health Organization classification of tumours: pathology and genetics of tumours of the digestive system.

[2] Fujii T, Rembacken BJ, Dixon MF, Yoshida S, Axon AT. Flat adenomas in the United Kingdom: are treatable cancers being missed? Endoscopy. 1998;30:437-43. 10.1055/s-2007-1001304 9693889

[3] Rembacken BJ, Fujii T, Cairns A, Dixon MF, Yoshida S, Chalmers DM, et al. Flat and depressed colonic neoplasms: a prospective study of 1000 colonoscopies in the UK. Lancet. 2000;355:1211-14. 10.1016/s0140-6736(00)02086-9 10770302

[4] Saitoh Y, Waxman I, West AB, Popnikolov NK, Gatalica Z, Watari J, et al. Prevalence and distinctive biologic features of flat colorectal adenomas in a North American population. Gastroenterology. 2001;120:1657-65. 10.1053/gast.2001.24886 11375947

[5] Tsuda S, Veress B, Tóth E, Fork FT (2002). Flat and depressed colorectal tumours in a southern Swedish population: a prospective chromoendoscopic and histopathological study. Gut.

[6] Moriichi K, Fujiya M, Sato R, Watarai J, Nomura Y, Nata T (2012). Back-to-back comparison of auto-fluorescence imaging (AFI) versus high resolution white light colonoscopy for adenoma detection. BMC Gastroenterol.

[7] Brewer MA, Johnson K, Follen M, Gershenson D, Bast R (2003). Prevention of ovarian cancer: intraepithelial neoplasia. Clin Cancer Res.

[8] Yaseen MA, Sakadžić S, Wu W, Becker W, Kasischke KA, Boas DA (2013). In vivo imaging of cerebral energy metabolism with two-photon fluorescence lifetime microscopy of NADH. Biomed Opt Express.

[9] Banerjee B, Renkoski T, Graves LR, Rial NS, Tsikitis VL, Nfonsam V (2012). Tryptophan autofluorescence imaging of neoplasms of the human colon. J Biomed Opt.

[10] Lin B, Urayama S, Saroufeem RM, Matthews DL, Demos SG (2011). Endomicroscopy imaging of epithelial structures using tissue autofluorescence. J Biomed Opt.

[11] Nakano K, Harada Y, Yamaoka Y, Miyawaki K, Imaizumi K, Takaoka H (2013). Precise analysis of the autofluorescence characteristics of rat colon under UVA and violet light excitation. Curr Pharm Biotechnol.

[12] Vishwasrao HD, Heikal AA, Kasischke KA, Webb WW (2005). Conformational dependence of intracellular NADH on metabolic state revealed by associated fluorescence anisotropy. J Biol Chem.

[13] Mayevsky A, Rogatsky GG (2007). Mitochondrial function in vivo evaluated by NADH fluorescence: from animal models to human studies. Am J Physiol Cell Physiol.

[14] Graves EE, Ripoll J, Weissleder R, Ntziachristos V (2003). A submillimeter resolution fluorescence molecular imaging system for small animal imaging. Med Phys.

[15] Sackmann M (2000). Fluorescence diagnosis in GI endoscopy. Endoscopy.

[16] Römer TJ, Fitzmaurice M, Cothren RM, Richards-Kortum R, Petras R, Sivak MV Jr, et al: Laser-induced fluorescence microscopy of normal colon and dysplasia in colonic adenomas: implications for spectroscopic diagnosis. Am J Gastroenterol. 1995;90:81-7. 7801955

[17] Zonios GI, Cothren RM, Arendt JT, Wu J, Van Dam J, Crawford JM, et al: Morphological model of human colon tissue fluorescence. IEEE Trans Biomed Eng. 1996;43:113-22. 10.1109/10.481980 8682522

[18] Gulledge CJ, Dewhirst MW (1996). Tumor oxygenation: a matter of supply and demand. Anticancer Res.

[19] Moriichi K, Fujiya M, Sato R, Nata T, Nomura Y, Ueno N (2011). Autofluorescence imaging and the quantitative intensity of fluorescence for evaluating the dysplastic grade of colonic neoplasms. Int J Colorectal Dis.

[20] Takehana S, Kaneko M, Mizuno H (1999). Endoscopic diagnostic system using autofluorescence. Diagn Ther Endosc.

[21] Van den Broek FJ, Fockens P, Van Eeden S, Kara MA, Hardwick JC, Reitsma JB, et al. Clinical evaluation of endoscopic trimodal imaging for the detection and differentiation of colonic polyps. Clin Gastroenterol Hepatol. 2009;7:288–95. 10.1016/j.cgh.2008.10.025 19168154

[22] Imaizumi K, Harada Y, Wakabayashi N, Yamaoka Y, Konishi H, Dai P (2012). Dual-wavelength excitation of mucosal autofluorescence for precise detection of diminutive colonic adenomas. Gastrointest Endosc.

[23] Seo M-W, Sawamoto T, Akahori T, Lui Z, Iida T, Takasawa T (2012). A low-noise high-dynamic-range 17-b 1.3-megapixel 30-fps CMOS image sensor with column-parallel two-stage folding-integration/cyclic ADC. IEEE Trans Electron Devices.

[24] Seo M-W, Suh S, Iida T, Takasawa T, Isobe K, Watanabe T (2012). A low-noise high intrascene dynamic range CMOS image sensor with a 13 to 19b variable-resolution column-parallel folding-integration/cyclic ADC. IEEE J Solid-State Circuits.

[25] Participants in the Paris Workshop (2003). The Paris endoscopic classification of superficial neoplastic lesions: esophagus, stomach, and colon. Gastrointest Endosc.

[26] Nishihara R, Wu K, Lochhead P, Morikawa T, Liao X, Qian ZR (2013). Long-term colorectal-cancer incidence and mortality after lower endoscopy. N Engl J Med.

[27] Kudo S, Kashida H, Nakajima T, Tamura S, Nakajo K (1997). Endoscopic diagnosis and treatment of early colorectal cancer. World J Surg.

[28] Selby JV, Friedman GD, Quesenberry CP, Weiss NS (1992). A case–control study of screening sigmoidoscopy and mortality from colorectal cancer. N Engl J Med.

[29] Lieberman DA, Weiss DG, Bond JH, Ahnen DJ, Garewal H, Chejfec G (2000). Use of colonoscopy to screen asymptomatic adults for colorectal cancer. Veterans Affairs Cooperative Study Group 380. N Engl J Med.

[30] Matsuda T, Saito Y, Fu KI, Uraoka T, Kobayashi N, Nakajima T (2008). Does autofluorescence imaging videoendoscopy system improve the colonoscopic polyp detection rate?—a pilot study. Am J Gastroenterol.

[31] Machida H, Sano Y, Hamamoto Y, Muto M, Kozu T, Tajiri H, et al. Narrow-band imaging in the diagnosis of colorectal mucosal lesions: a pilot study. Endoscopy. 2004;36:1094–98. 10.1055/s-2004-826040 15578301

[32] Su MY, Hsu CM, Ho YP, Chen PC, Lin CJ, Chiu CT. Comparative study of conventional colonoscopy, chromoendoscopy, and narrow-band imaging systems in differential diagnosis of neoplastic and nonneoplastic colonic polyps. Am J Gastroenterol. 2006;101:2711–16. 10.1111/j.1572-0241.2006.00932.x 17227517

[33] Tischendorf JJ, Wasmuth HE, Koch A, Hecker H, Trautwein C, Winograd R. Value of magnifying chromoendoscopy and narrow band imaging (NBI) in classifying colorectal polyps: a prospective controlled study. Endoscopy 2007;39:1092–96. 10.1055/s-2007-966781 18072061

[34] Hirata M, Tanaka S, Oka S, Kaneko I, Yoshida S, Yoshihara M, et al. Magnifying endoscopy with narrow band imaging for diagnosis of colorectal tumors. Gastrointest Endosc. 2007;65:988–95. 10.1016/j.gie.2006.07.046 17324407

[35] East JE, Suzuki N, Saunders BP (2007). Comparison of magnified pit pattern interpretation with narrow band imaging versus chromoendoscopy for diminutive colonic polyps: a pilot study. Gastrointest Endosc.

[36] Chiu HM, Chang CY, Chen CC, Lee YC, Wu MS, Lin JT, et al. A prospective comparative study of narrowband imaging, chromoendoscopy, and conven conventional colonoscopy in the diagnosis of colorectal neoplasia. Gut. 2007;56:373–79. 10.1136/gut.2006.099614 PMC1856788 17005766

[37] Rastogi A, Bansal A, Wani S, Callahan P, McGregor DH, Cherian R, et al. Narrow-band imaging colonoscopy – a pilot feasibility study for the detection of polyps and correlation of surface patterns with polyp histologic diagnosis. Gastrointest Endosc. 2008;67:280–86. 10.1016/j.gie.2007.07.036 18155210

[38] Rex DK, Ahnen DJ, Baron KP, Burke CA, Burt RW, Goldblum JR (2012). Serrated lesions of the colorectum: review and recommendations from an expert panel. Am J Gastroenterol.

[39] Kagawa K, Zhang B, Seo MW, Kawahito S, Kominami Y, Yoshida S (2013). Dual-band multi-aperturenhanced redox imaging of colonic adenomas for endoscopes with a high-performance CMOS imager. Conf Proc IEEE Eng Med Biol Soc.

